# Key informant perspectives on the challenges and opportunities for using routine health data for decision-making in Senegal

**DOI:** 10.1186/s12913-021-06610-1

**Published:** 2021-06-22

**Authors:** Pierre Muhoza, Haneefa Saleem, Adama Faye, Ibrahima Gaye, Roger Tine, Abdoulaye Diaw, Alioune Gueye, Almamy Malick Kante, Andrea Ruff, Melissa A. Marx

**Affiliations:** 1 Department of International Health, Johns Hopkins Bloomberg School of Public Health, Baltimore, MD USA; 2 Institut de Santé et Développement, Université Cheikh Anta Diop de Dakar, Dakar, Senegal; 3 Faculté de Médecine, de Pharmacie et d’Odontologie, Université Cheikh Anta Diop de Dakar, Dakar, Senegal; 4 Direction de la Planification, de la Recherche et des Statistiques/ Division du Système d’Information Sanitaire et Social, Ministère de la Santé et de l’Action Sociale (MSAS), Dakar, Senegal; 5 Programme National de Lutte Contre le Paludisme, Ministère de la Santé et de l’Action Sociale (MSAS), Dakar, Senegal

**Keywords:** Routine health information systems, Data quality, Data use, Senegal, Malaria, Tuberculosis, HIV/AIDS, Routine data

## Abstract

**Background:**

Increasing the performance of routine health information systems (RHIS) is an important policy priority both globally and in Senegal. As RHIS data become increasingly important in driving decision-making in Senegal, it is imperative to understand the factors that determine their use.

**Methods:**

Semi-structured interviews were conducted with 18 high- and mid-level key informants active in the malaria, tuberculosis and HIV programmatic areas in Senegal. Key informants were employed in the relevant divisions of the Senegal Ministry of Health or nongovernmental / civil society organizations. We asked respondents questions related to the flow, quality and use of RHIS data in their organizations. A framework approach was used to analyze the qualitative data.

**Results:**

Although the respondents worked at the strategic levels of their respective organizations, they consistently indicated that data quality and data use issues began at the operational level of the health system before the data made its way to the central level. We classify the main identified barriers and facilitators to the use of routine data into six categories and attempt to describe their interrelated nature. We find that data quality is a central and direct determinant of RHIS data use. We report that a number of upstream factors in the Senegal context interact to influence the quality of routine data produced. We identify the sociopolitical, financial and system design determinants of RHIS data collection, dissemination and use. We also discuss the organizational and infrastructural factors that influence the use of RHIS data.

**Conclusions:**

We recommend specific prescriptive actions with potential to improve RHIS performance in Senegal, the quality of the data produced and their use. These actions include addressing sociopolitical factors that often interrupt RHIS functioning in Senegal, supporting and motivating staff that maintain RHIS data systems as well as ensuring RHIS data completeness and representativeness. We argue for improved coordination between the various stakeholders in order to streamline RHIS data processes and improve transparency. Finally, we recommend the promotion of a sustained culture of data quality assessment and use.

## Background

Ideally, in a well-performing health system, quality routine health information system (RHIS) data should be used to guide decision-making processes [1]. In addition to improving program efficiency and equity in resource allocation, data-informed decision-making should theoretically contribute to a culture of accountability and transparency if a given course of action is taken based on a full assessment of all available quality data. However, the existing literature suggests that decision-making is a complex process that can be influenced by a multitude of factors such that the reliance on available health data as the basis for decisions does not take precedence [2–4].

Multiple barriers and facilitators can influence individuals and organizations to use data effectively in the decision-making process. These include poor data quality [5–7]; insufficient skills in core competencies of data use [7–10]; poor RHIS design [11]; inadequate access to relevant data for decision-making [6, 10]; limited interaction between data producers and data users that leads to a disconnect in terms of data demand and use [6, 12, 13]; and institutional factors that determine authority structures and influence decision-making [12, 14].

Though an increasing body of evidence from Low and Middle Income Countries (LMICs) has contributed to our understanding of the determinants of RHIS data use, there is a scarcity of up-to-date peer-reviewed evidence specific to the Senegal context [15, 16]. Previous studies that have sought to address the issue of data use in Senegal either did not focus on RHIS data specifically, or emphasize country-specific issues sufficiently [15], limited themselves to issues specific to family planning and reproductive health [16] or focused on health system governance issues related to data production [17]. Nonetheless, results from these studies suggest that common data use challenges in Senegal include limited access of relevant data to the appropriate user due to poor data sharing practices or insufficient digitization of data, as well as barriers related to communication technologies for storing and sharing data. Findings from the few studies that have attempted to evaluate the quality of RHIS data or the feasibility of using RHIS data for decision-making have shown that RHIS data quality in Senegal is highly variable across levels of the health system, across disease programs and across indicators even within a single program [18–20]. Inaccurate or incomplete records and delays in facility reporting have been documented as challenges that may influence the quality and hence, use of RHIS data in Senegal [18, 20].

Senegal is an LMIC in West Africa that has recently undergone dynamic changes in the health sector. International funding agencies have supported joint partnerships with the Senegal Ministry of Health (MoH) to shape health service delivery and RHIS implementation. The resulting inflow of funds and increased calls for accountability coupled with an evolving epidemiologic profile have led to profound changes in intervention strategies and in monitoring and evaluation (M&E) processes. In particular, the malaria, HIV/AIDS and tuberculosis (TB) programmatic areas have received a considerable amount of attention given the epidemiologic importance of the diseases, their far-reaching socioeconomic consequences and the complexity of interventions needed to sustain gains made [21–24]. A wide range of public sector, private sector and civil society actors operates through complex partnerships at the community- and facility-levels to ensure the equitable coverage of HIV, malaria and TB health services and commodities. Through its Division of Social and Health Information Systems (DSISS), the MoH has continued to strengthen M&E capabilities by phasing out paper-based reporting and introducing the District Health Information System (DHIS2) [25]. This open source system was piloted in 2014 and underwent gradual rollout across Senegal’s 76 health districts until it was declared the national RHIS in 2016. Attention has been dedicated to the implementation of the system and there is a growing imperative to not only ensure that the RHIS data collected are of high quality, but also that they are used for decision-making. In response, the most recent individual strategic plans set forth by the MoH’s national programs for malaria, TB and HIV have all included the strengthening of RHIS with an emphasis on using RHIS data for decentralized planning, improving supply chain management, improving the quality of interventions and expanding community-based interventions [23, 24, 26].

This study aims to examine the current RHIS data use environment in Senegal, identify the barriers and facilitators to RHIS data use, as well as opportunities for moving the RHIS data use agenda forward. Since the few existing studies in Senegal exploring data use were conducted prior to the aforementioned transformative changes, did not specifically focus on RHIS and did not address perspectives from the civil society [15–17], we aim to fill an important gap in the literature.

## Methods

### Study design, setting and participants

We conducted this qualitative study between January 2019 and November 2019. We used key informant interviews in order to obtain a detailed understanding of the routine data use environment in Senegal. Individuals who had knowledge of and experience with implementing and/or evaluating HIV, tuberculosis or malaria programs in Senegal were selected purposively based on institutional affiliation to ensure that appropriate informants would provide rich study data. Inclusion criteria for this study were age > 18, willingness to provide a written consent for an interview and being an employee of an organization involved in malaria, HIV or TB control. All respondents were employed in organizations that were current or past recipients of grants awarded directly or indirectly by a single donor organization for HIV, TB or malaria. Since we were interested in RHIS data use processes at the national level, we restricted the sampling frame to individuals working at the central level of the health system or individual organizations.

We defined two categories of key informants. The first category included high-level decision-makers (DM) defined as those individuals in a position to make decisions on policies, operational protocols, project designs, and resource allocation such as program directors or coordinators, program managers and program officers. The second category included mid-level personnel (MP) such as monitoring and evaluation officers or analysts. In their midlevel capacity, they constitute an integral part of the data dissemination pathway mediating data producers from the lower levels of the health system and the decision-making end users.

Stakeholder analyses conducted alongside researchers from the *Université Cheikh Anta Diop de Dakar* (UCAD) who had strong knowledge of the programmatic functioning across the HIV, TB and malaria fields in Senegal enabled the development of a list of potential respondents. Ultimately, study respondents included informants from divisions of the Ministry of Health, nongovernmental organizations with local and international scopes, and the civil society with representation from a faith-based organization and a community-based organization that work to achieve access to health care for vulnerable and key populations. Overall 12 decision-makers and 11 mid-level personnel across these organizations were invited to participate in the study. Invitations for interviews ceased when no further themes could be identified (i.e, data saturation) [27].

### Data collection

Individual-level key informant interviews were conducted in French and took between 30 and 45 min. All interviews took place in person in the private offices of the respondents. Interviews were audio-recorded, transcribed verbatim and proofread by a researcher from Johns Hopkins University (JHU) with native fluency in French and graduate training in qualitative research methods.

We used a semi-structured guide for the key informant interviews to enable the exploration of a consistent set of questions while at the same time providing the flexibility to probe on themes specific to the key informant. To develop questions for the interview guide, we adapted the MEASURE Evaluation tool designed to provide a rapid assessment of data demand and constraints on data use at various levels of the health system [28]. Key informants were asked questions about the decision-making process and information flow in their organization; their attitudes towards routine data and their quality; the barriers and facilitators influencing the use of routine data; their perceptions of the existing best practices as they relate to routine data and the recommendations to improve the use of routine data.

Prior to the data collection activities, three JHU faculty members and two UCAD faculty reviewed the interview guide to ensure rigor and appropriateness for the study. Although the core questions remained consistent throughout the data collection activities, probes were modified as we gained understanding of the Senegalese programmatic context and to follow-up on specific areas of technical expertise of the respondents.

### Data analysis

We conducted a framework analysis starting with deductive coding and grouping of transcripts in relation to key informant categories: decision-makers versus midlevel personnel; stakeholder affiliation and disease of focus. The codes used in the deductive coding phase were informed by a review of existing academic and grey literature in the field of routine data quality and data use [16, 29–32]. This coding phase was followed by inductive coding to enable a more data-driven approach that allowed the development of emergent codes specific to the Senegal data use environment. Similar codes were then grouped into categories to simplify comparison of transcripts and identification of patterns. The iterative processes of coding, categorization and cross-case analysis were conducted using Atlas.ti 7.5 and Excel. These analytic processes were conducted by author PM under the supervision of authors HS and MM. Interview transcripts were analyzed in French and select quotes illustrating key themes were translated to English.

**Fig. 1:**
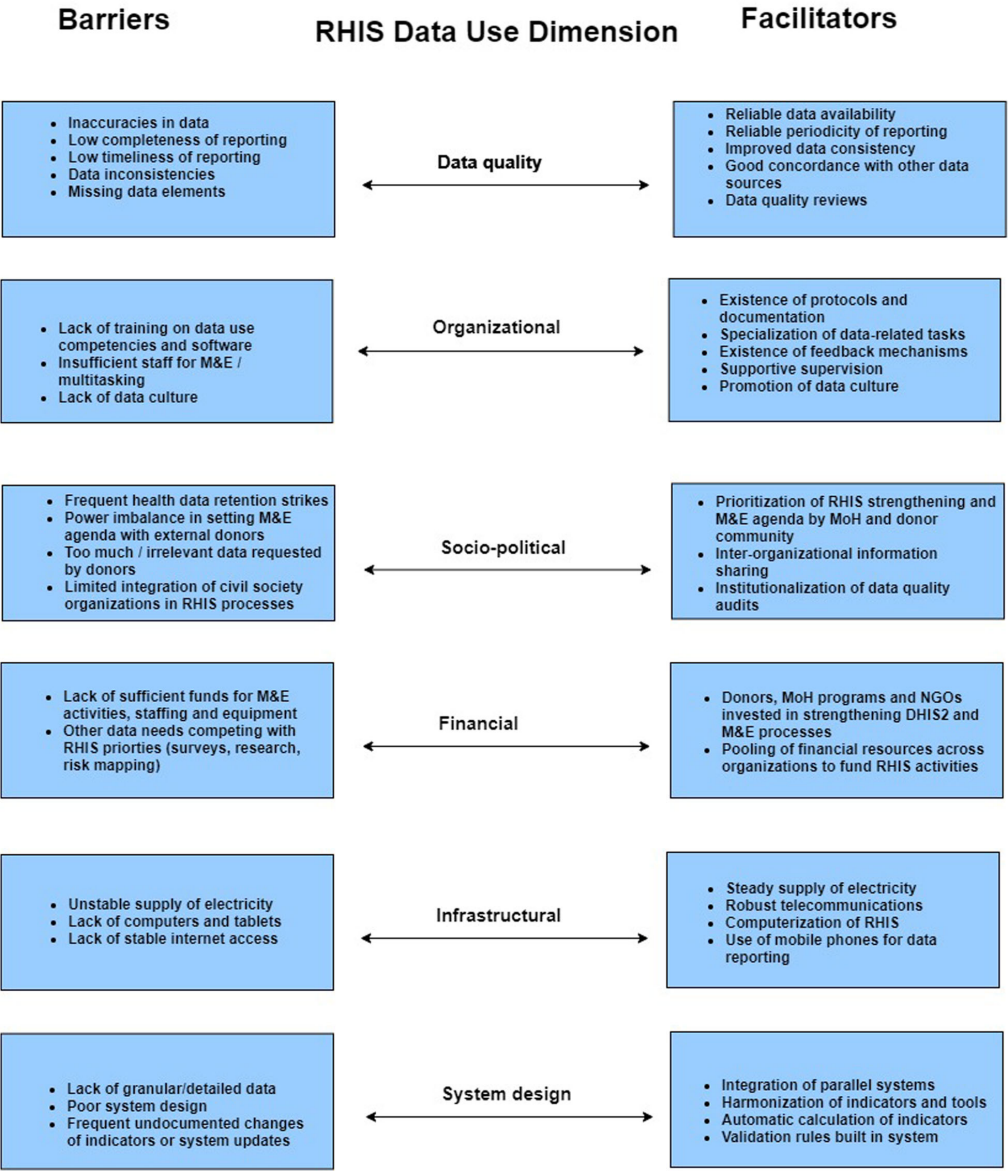
Summary of RHIS data use barriers and facilitators identified in Senegal

Six main emergent data use dimensions (Fig. 1) guided further analysis and interpretation. In the data quality dimension, we considered the influence of frequently-cited characteristics that determine data usefulness in programmatic settings: completeness, timeliness, accuracy and consistency with other data sources [33]. In the organizational dimension, we examined the organizational structures, rules, values and practices influence the context within which the users access, disseminate and use routine data [31, 32]. The sociopolitical dimension allowed us to consider the influence of the sociopolitical system within which RHIS data are used, policies relevant to data use in Senegal, and the role of the relationships among the various stakeholders. We examined factors influencing the funding and prioritization of RHIS data and processes in the financial dimension. In the infrastructural dimension, we examined the basic structures and material supplies required for RHIS processes. In the system design dimension, we considered factors that define the RHIS interface, data, user and system behavior to address data needs for decision-making [11].

### Ensuring quality and rigor

Following analysis of the collected qualitative data, we conducted respondent validation procedures in order to strengthen the validity and credibility of research findings. Emergent themes were summarized and returned to respondents by an email containing a link to an online survey (Qualtrics). The survey included prompts inviting respondents to reflect critically on the research findings and to highlight any instances of incorrect interpretations of facts. The survey also provided respondents with an additional opportunity to offer recommendations for improving data use. We treated input from respondents regarding the summary and preliminary interpretations as additional data. A maximum of three email reminders were sent to non-responders over the course of 4 weeks requesting their participation.

## Results

### Characteristics of key informants

Eighteen key informants (15 male, 3 female) were purposively recruited (Table 1) from ten different organizations. Five key informants (3 decision-makers, 2 mid-level personnel) declined to participate in the study due to availability constraints. The response rate for the respondent validation procedure was 61% (11 respondents).

**Table 1:** Characteristics of key informants

	Stakeholder type Number of informants N(18)	
	Decision-makers	Mid-level personnel
**Stakeholder affiliation**		
MoH Programs	3	3
International NGOs	3	2
National NGOs / CSOs	3	4
**Disease focus**^a^		
HIV/AIDS	6	7
Malaria	5	3
Tuberculosis	2	4
**Number of years in position (or working in field)**	**Range**: 0.5–13 years **Median**: 6.25 years	

^a^Several stakeholder organizations have experience with more than one of the disease programs. In this study, disease focus was determined based on respondent-stated focus of organization efforts. Thus the totals in this particular section of the table do not correspond to the total N

*NGO* Non-Governmental Organization

*CSO* Civil Society Organization

### Quality of RHIS data

#### Timeliness and completeness of reporting

There was broad consensus that the quality of RHIS data poses a major challenge in their use for decision-making. Among the consistently cited reasons for the poor quality of data was the lack of complete and timely availability of data. Although respondents indicated the rates of complete and timely reporting have improved over the past few years, there was agreement that the limited availability of routine data on a timely and reliable basis frequently hampers decision-making. Other factors affecting data completeness that were commonly cited by respondents included the fact that data from some health facilities such as pharmacies, private and referral hospitals are not captured by the DHIS2. This is illustrated by the quote below from an MoH respondent.

“*… The private [facilities] are configured in the DHIS2 platform but unfortunately their data are not captured. Also the data from hospitals poses problems*”

- MP#1

#### Accuracy and completeness of data elements

The completeness and accuracy of data elements submitted in program data reports and into the DHIS2 were also reported to be common problems that reduce the reliability of data. Informants often attributed the occurrence of these errors to individual-level circumstances during data collection. The general perception was that the heavy workload and fatigue among operational-level data collectors or their lack of understanding of the importance of certain data elements contributed heavily to data entry errors.

“*We also have problems with the completeness of the filling of the [data collection] tools. … Routine data cannot be used when the register is not filled in completely and correctly.* ”

- MP#5

### Organizational factors

Insufficient and poorly trained human resources for data tasks were among the most consistently cited organizational barriers to the use of routine data irrespective of the different categories of respondents. These were cited to be problems both at central levels and operational levels of the health system. Conversely, organizations that had sufficient staff and established mechanisms for regular training generally highlighted their importance in ensuring improvements in data quality and data use.

*“I must say that we have a good experience in using routine data. From the point of data collection to the use of data for decision-making. … Staff are adequately trained because we have M&E procedure manuals on surveillance guidelines on which people are regularly retrained.”*

- DM#7

There was a consensus among respondents that having insufficient staff for data-related tasks leads to inefficiency since the few available staff have to multitask. Several informants indicated that specializing data tasks either by delegating people who are specifically trained to handle data or having a dedicated time for data-related tasks could improve the efficiency.

*“The person that deals with data at the facility-level has many other tasks. He is not a data specialist. Usually the primary care supervisor is also responsible for data management. I think we must move towards specializing people in the management of facility data... Because if there was “a data guy” that routinely manages data for all programs for each facility, I think that could definitely improve data management”.*

- MP#2

“*There are some districts that reserve 2 days to collect programmatic data …. to achieve good reporting timeliness and completeness... And if, for example, all 76 districts did this and for all the programs, we would not have a problem.”*

- DM#2

### Socio-political factors

#### Health data retention strikes

Study respondents indicated that the frequent health data retention strikes coordinated by Senegal’s health worker unions posed a major impediment to the availability and thus use of RHIS data. The data retention strikes require that health workers on every level of the national health system withhold from the Senegalese Ministry of Health routine patient data. Some respondents indicated that to mitigate the data availability challenges and ultimately meet their data reporting obligations to donors, they had to resort to strategies that include creating and financing their own parallel data collection systems or relying on personal relationships with facility-level data managers to obtain routine data via unofficial channels during the data retention strikes. One respondent provided information exemplifying the frequency and consequences of the health data retention strikes.

*“Almost every year, you can go 6 months without data. Over the past year, we went 9 months without data.... And that's a recurring problem. Sometimes you end up with 10 year old projects that suddenly fall apart because of data retention... We once lost a project worth billions [in local currency] because of that. We didn't have any data to give to our donor. Because at the end of the day, the donors will ask you for the data... If you can't provide your report, well then your donor will take his money.”*

- DM#5

#### Role of the donors

We noted that the dynamics of the relationship between the individual organizations and their donors (whether local or foreign) were important in shaping RHIS and data use practices. For example, we found that data demand by donors had a strong influence on the type and amount of data collected by grant recipient organizations and by the broader health system. There were several reports of donors preferring to support parallel data collection systems that are separate from the national RHIS in order to either obtain data specific to donor needs or to circumvent data quality concerns. The presence of multiple parallel systems combined with donor requests for large amounts of data irrespective of their relevancy for decision-making often created a heavy burden for staff working at lower levels of the data use chain ultimately impacting the quality of the reported data.

“*Nowadays, too much information that is not even used annually is expected from the routine system… There may be 5 to 10 registers to complete a day. And often the quality of completion then becomes a problem for us... and often the projects that have funding are the ones who dictate the law... And sometimes it is data that the [health] system itself does not even use*”

- DM#4

#### Health system structure and data use

Within the sociopolitical dimension, we also considered how the decentralized structure of the Senegal health system might influence the production, transmission and use of RHIS data. Informants reported consistently that lower levels of data use and inattention to data quality at district- and facility-levels contributed heavily to the poor quality data reaching the central level. Many respondents for example indicated that although districts are required to conduct periodic data quality assessments (DQAs), few of them actually conduct them on a regular basis.

“*… We know that in reality [the quarterly DQA] is not done and that's a problem. I think the [DQAs] have to be institutionalized… Even when there is no donor financing, the districts should go out and do the audits…That way the regularity [of the DQAs] is respected*”

- MP#6

Authority structures and the dynamics of the data producer – data user relationships were often said to influence the regularity of DQAs and use of RHIS data at lower levels of the health system. For example, when a high-level MoH respondent was asked why some of the recommendations she was providing were not already in place given their appropriateness, she said:

“*We coordinate from the central level but we do not produce the data. We are just data collectors. The real data producers are the districts... I can provide advice [to the chief nurses and medical officers]. But I am not their leader... Whatever I have to tell them, I have to go through the chief district medical officer since he is their direct leader. On the other hand, I can for example propose directives to my data managers.”*

- DM#2

#### Institutional relationships: sharing data and data use experiences across organizations

Respondents generally observed that cross-organizational sharing of information including RHIS datasets and data use experiences were among the best existing practices in Senegal. Several respondents cited the publication of surveillance bulletins, organization of regular data review meetings and coordination workshops (instances during which health facility leaders discuss facility performance and the quality of their data) as factors that promote accountability and maintain the focus on the importance of data.

In general, there was consensus that the existing channels for sharing data across organizations were a facilitating factor to data use. Respondents often linked fluid sharing of RHIS data to concepts such as improving efficiency, accountability, transparency, promotion of a shared data culture and RHIS data reliability. Notably, though CSO informants appreciated the existing data sharing efforts, many nonetheless felt some tension in their coordination with public sector actors. More specifically, some felt sidelined from the national conversations about data collection processes since they are only invited in data coordination meetings only to share their data for validation purposes while others expressed frustration about the lack of approvals to access the DHIS2 platform. Many CSO informants thus emphasized the need for increased involvement in national conversations about data-related processes, improved access to the national RHIS and improved partnerships with the public health sector actors of different levels.

*“The civil society does not have access to DHIS2.... I remember we were in a meeting…when the discussion about access to the system came up. Things became complicated because of medical regions. They represent the health system at the regional level and sometimes it gets complicated because they see civil society as competitors whereas we're just there to support the system.*”

- MP#7

### Financial factors

Financial considerations emerged as important determinants of RHIS data use, particularly among decision-makers. Despite the political will generated by the engagement of external donors and the MoH to mobilize resources for RHIS strengthening, respondents frequently cited the lack of sufficient funding as an impediment to the optimal use of routine data in Senegal. Lack of funding was reported to be a reason for not being able to employ adequate staff for M&E activities, implementing crucial data-related activities (i.e. data quality assessments, training activities and data use workshops) and ensuring the adequacy of data-related equipment (i.e. computers, tablets, software). Conversely, donor funding or pooling of resources across organizations provided resources necessary to support data quality and data use logistics.

The recurrent theme among informants was that there were many competing data priorities and that presently, there was a considerable mismatch between available financial resources and needs. For example, informants often stressed the importance of balancing the financing of RHIS-related activities such as (DQAs, trainings, workshops and supervisions) against financing other activities that produce data enabling the contextualization and better use of RHIS data. Examples included the collection of qualitative data as well as the conduct of survey, risk mapping and research activities. Of note, the importance of DQAs and investing in them appeared to be more salient to midlevel personnel as compared to decision-makers. On the other hand, compared to midlevel personnel, decision-makers appeared to value more the use of survey data for decision-making and hence increased investments in the collection of the data. For example, an informant involved in TB control efforts said:

“*The [HIV & malaria programs] conduct periodic surveys. But for us, it may even be the nature of our disease as we can’t go around asking everyone [for sputum samples]. The appropriate survey would be based on radiographs. Yet, this is extremely expensive...I say routine data are good, but surveys are also very important. Surveys allow comparison with routine data to see if we're on the right track or not*”

- DM#2

### Infrastructural factors

In general, key informants said that the shift from paper-based reporting to a more computerized approach had significantly improved the way they collect, transmit and use data. The use of computers, tablets and phones to enter and transmit routine data was a commonly cited facilitator to the availability of quality data. Conversely, the lack of the aforementioned resources combined with weak telecommunications and unstable supply of electricity were frequently cited to be significant barriers to data availability. Furthermore, informants suggested that the availability of these resources influenced the motivation of individual workers at peripheral levels of the health system to perform their RHIS tasks. For example, one respondent said:

“*We need to support and motivate [the frontline staff who manage the DHIS2 platform]... They are asked to maintain the DHIS2 platform. If you don't have a working [internet] connection, a computer or enough [office] space to manage things, how can that be motivating?”*

- MP#5

### RHIS system design factors

The most important RHIS design factors were 1) the harmonization of indicator definitions and primary data collection tools across the different vertical health programs Senegal, 2) the integration of program-specific tools onto a single platform and the automation of data management processes such as data entry validation and 3) the automatic calculation of indicators. Informants also reported that storing data collected from different programmatic areas in one place reduced redundancies in collecting and transferring data. Overall, this improved organizational efficiency. Despite the successes in the harmonization of M&E tools, respondents noted two main challenges. The first one was that there were frequent updates to indicator definitions in response to changing disease control strategies and sometimes these updates were not well-documented or well-communicated to lower levels of the health system. The second challenge noted by respondents was that harmonization efforts were not well coordinated across levels of the health system. Poor coordination among partners intervening at the community-level contributed to this perception.

“*Every time we make a small programmatic change, people want to go back and revise the tools. And that's what brings about the constant change… I think we would benefit from having a system … of technical notes that document the updates in M&E guidelines…Very often you go to the regions, you meet the agents. Some tell you [of changes] that are not based on any document. And often, it is someone who has been to a central-level meeting who then comes back to spread information word of mouth. That is a harmful element.”*

- DM#4

“*At the community level, we are overloaded with tools and information…Our partners really need to harmonize with what we have and the data we want to collect so that we can all align ourselves with the needs [and] tools of the MoH…and avoid overburdening community [health] workers*”

- MP#9

Though informants generally valued RHIS’ ability to provide useful aggregate information, many lamented the loss of granularity lost in the aggregation. In particular, civil society informants expressed frustration with DHIS2’s inability to distinguish individual contributions by organizations. The system’s categorization of data elements prevented organizations from being able to validate reported data, address inconsistencies and highlight their contributions to increase organizational visibility. They often indicated that addressing these issues would help promote accountability, attribution of effort in a crowded field of implementing partners and also enable mechanisms to validate data quality.

“*Often in meetings we are told that, in a given district, there are no populations [of men who have sex with men] or data on key populations while we are aware that we have worked [with them] and that the data has been transmitted to the district-level. However, in the health system, they simply record the data as “people living with HIV”... and then we lose our data. It is not a loss per se. Nevertheless, these data do not reflect the reality. It is true that they are people living with HIV but from quite specific groups.”*

- DM#6

Virtually all informants working in the HIV field pointed to RHIS’ inability to prevent the double counting of individuals accessing services at multiple sites, from different providers or in different geographic locations as a major problem. Many said that this contributed to poor data quality and reduced trustworthiness of the data. To address this problem, informants from the NGOs and CSOs encouraged the adoption and integration of unique personal identifiers into the RHIS to reduce the duplication of efforts.

“*We should solve the problem of unique identification codes to avoid duplication. We operate in a multi-program context where people work in the same area to reach the same people.* ”

- DM#8

We also identified data granularity issues salient to informants working in malaria control. Informants generally expressed a need for more periodic and spatially granular data. Most found the national RHIS data to be inadequate since they do not allow the distinction between malaria cases resulting from local transmission versus imported. Yet this is an important consideration for Senegal’s goal to eliminate malaria by 2030.

“*The data in the registers cannot allow you to measure whether you have a local incidence or an administrative incidence? Is this case imported? Is this case not imported? Register data do not provide this indicator for decision-making relative to this issue*”

- DM#5

## Discussion

Informants participating in our study perceived RHIS data as crucial in setting programmatic priorities and driving strategies in the Senegal context. Nonetheless, there was a general consensus that though there had been considerable investments in RHIS and improvements in data availability, the full potential of RHIS data for decision-making is yet to be achieved. As others have previously observed, we found that the quality of available RHIS data was an important factor in determining both the value that data users place on RHIS data and the likelihood of use for decision-making [5–8]. Findings further show that several upstream factors interact in a complex fashion to ultimately influence the quality of data available and the individual determinants of decision-making. Although there were commonalities in these determinants across informants, we expect that in practice, the relative individual importance of these factors in determining data quality or data use may vary by disease program and organization. Here, we discuss the major points relevant to all cadres of informants and offer our perspective on the five key priorities for action to strengthen the use of RHIS data for programmatic decision-making in Senegal.

### Priorities for action

#### Ensure uninterrupted surveillance and continuity of RHIS data reporting activities

Our findings highlighted key factors related to health system governance that ultimately influenced the continuity of surveillance activities and by extension the availability of RHIS data as well as its use for decision-making. In particular, the health data retention strikes that were consistently cited by respondents had socio-political and financial determinants. Health worker strikes have been previously described in various countries along the development continuum and the literature points to a wide array of strategies used by the striking health workers to obtain the desired concessions [34–36]. The strikes in Senegal are unique in that they constitute a rare documented case of specifically withholding health data as leverage in negotiations with authorities. As reported by the respondents in our study and previously described elsewhere, the retention of health data retention during strikes has been a persistent problem in Senegal since at least 1996 [17, 37, 38]. Between 1997 and 2000 for instance, the health worker strikes prevented the collection of routine data [17]. The malaria RHIS data collected during the 2010–2012 period are generally understood to be of poor quality since they were backfilled in March 2013 after the resolution of multiyear disputes [39]. Unfortunately, the frequency of the strikes has impacted the reliability of RHIS data to the extent that some implementation partners reportedly prefer using the Senegal Demographic and Health Survey (DHS) data instead [40].

There are no simple solutions for health worker strikes as their causes are typically far-ranging and intertwined with complex political as well as socio-economic determinants. Solutions proposed in recent literature have included calls for multisectoral coordination to improve the working conditions of the health workers and building resilient health systems capable of maintaining function even in times of crises [41, 42]. Addressing the different infrastructural and financial challenges that the health system workforce faces is a sound preventive strategy for tackling data strikes and building health system resilience. In response, some external donors have developed mechanisms of support that specifically emphasize building resilience and sustainability through investments in human resources for health (HRH). For example, the Global Fund is increasingly encouraging grant applicants to demonstrate both the prioritization of HRH needs and the sustainability of relevant remuneration plans [22]. During the roll-out of the DHIS2 platform in Senegal, the Global Fund worked alongside GAVI and the World Bank to provide hardware and improve connectivity [43]. Likewise, the President’s Malaria Initiative (PMI) approach to health system strengthening emphasizes improving the organizational conditions that enable HRH to deliver quality health care while maintaining the RHIS [44].

#### Support and motivate HRH who maintain RHIS data systems

Ultimately, incentives and disincentives significantly influence individuals and thus overall organizational performance. Our findings suggest that in order to improve the quality of the RHIS data in Senegal, the implementation of incentive schemes addressing the financial and non-financial needs of frontline workers involved in RHIS activities will be crucial. Even though the study participants worked in the strategic levels of their respective organizations, they were generally cognizant of the importance of ensuring staff motivation at the peripheral levels by primarily improving working conditions. Indeed, previous studies have identified low pay and poor working conditions as major factors hindering the collection and use of data for decision-making [6]. To ensure that staff are motivated to perform their RHIS-related duties, it will therefore not only be necessary that they are paid their due wages regularly, but also that they are adequately trained and equipped for their tasks. Importantly, training of frontline staff should not only focus on the actual performance of their required tasks. Equally essential is communicating to the frontline workers how the quality of their individual contributions fit within the broader disease control agenda. Supervision, coaching and provision of regular feedback are well-studied approaches that may help staff improve their competence in RHIS-related duties [45–47]. A recent scoping review focusing mostly on African countries and summarizing the literature on interventions for improving RHIS data quality and data use found that successes at the health facility-level often involved a combination of technological interventions that facilitate RHIS-related duties (e.g., electronic data collection and management systems) with feedback and the capacity building mechanisms (training, coaching and supervision) [48]. In Senegal, a study that evaluated facility-level supply chain management for health commodities using available routine data, suggested a link between supervision and improved performance [20]. The study also suggested that the increased attention to the use of facility RHIS data led to improvements in data completeness.

It is important to recognize that effective management of the RHIS-related workload is also essential. It was clear from the respondents that staff at the lower levels of the health system experience high levels of data requests from programs. Furthermore, some facility-level personnel involved in the collection and dissemination of RHIS data have other competing priorities such that RHIS-related tasks are of low priority. A solution that has been successful in Botswana has included task-shifting initiatives that delegate RHIS-related duties to a dedicated cadre of specialized M&E professionals [49]. Elsewhere, some institutions of higher learning have even implemented graduate programs dedicated to specialized M&E trainees [50]. The suitability of these approaches in the Senegal context should be explored.

#### Ensure data completeness and representativeness of RHIS data

The present study also confirms results from previous studies in other countries showing that the completeness and timeliness of RHIS data reporting remain problematic [51]. Importantly, our study suggests that these aspects, in addition to data accuracy, may be the most important determinants of the confidence with which stakeholders may have in RHIS data. Data lose their utility if they are not reliably available for decision-making. They further lose credibility if stakeholders do not believe that they represent the reality in the field.

Difficulties with capturing routine data from private facilities certainly contribute to the perception that RHIS data lack completeness and representativeness. The private health sector in Senegal has experienced rapid growth in recent times and continues to play an important role in fulfilling health needs unmet by the public sector [52]. Nonetheless, enumerating and regulating private sector providers of HIV/AIDS and malaria services have proven to be difficult [52]. As the role of the private sector in health service delivery in the country has increased, so have the calls for increased reporting of activities in private sector facilities [43, 53]. Challenges in capturing RHIS data from non-reporting units such as private facilities are certainly not unique to Senegal and have been reported in other countries [54, 55]. As the DHIS2 system continues to gain prominence, it will be important for Senegalese policy-makers to explore mechanisms of incentivizing non-reporting actors to report their data to the national RHIS systematically. An immediate recommendation towards achieving this goal should therefore include discussions with actors in the private health sector on the basic reporting standards that private facilities should adhere to. Others have suggested approaches such as providing DHIS2 implementation packages to private sector health facilities that include training, standardized reporting tools and software support to expedite the uptake of the DHIS2 system in the private sector [54]. Ultimately, to achieve the much-desired RHIS data completeness, it will be crucial to integrate the private health sector fully into the national RHIS and in the enforcement of data reporting policies.

#### Strengthen partnerships and coordination with all stakeholders

Our study found that inconsistent and fragmented data collection processes impede efforts to evaluate needs and monitor impacts particularly at the community level, consistent with previous studies elsewhere in Africa [56, 57]. Senegal has developed one of the most successful models for community health in Africa. The rather differentiated model relies on five main community health provider cadres that provide specific interventions as part of integrated packages of health services: *agents de santé communautaires* and *relais communautaires* [regular community health workers - (CHWs)]; the *matrones; bajenu gox* and *dispensateurs de soins à domicile* (home-based care providers) [58]. In their duties to provide care, appropriate referrals for patients, manage stocks of health commodities and promote behavior change, the community health providers generate vast amounts of data. Since a wide range of actors with varying and sometimes competing objectives supports these community-level agents, there is a likelihood that they capture overlapping and redundant data. Study respondents from NGOs with programs operating at the community level suggested that there was an urgent need to ensure that data collection tools used by the community health providers are harmonized to ensure standardized data collection and reduce duplication of effort. Through partnerships with community-based organizations and the use of CHWs, the Senegal NGO / CSO sector fills key service provision gaps across the approximately 2000 health huts that cover 19% of the country’s population [52]. Given the sector’s importance in addressing unmet health needs Senegal, we recommend increased coordination within the sector and with the different development partners to ensure the realization of harmonization for M&E. The harmonization process may be best achieved under effective leadership of the MoH, active stakeholder engagement and the implementation of follow-up mechanisms for agreed recommendations [59]. Some collaborative efforts are already underway with the National Malaria Control Program working together with the DSISS to integrate data platforms developed and maintained by some NGO actors [43].

Truly strengthening partnerships for increased RHIS data use will require a more inclusive approach that emphasizes the data needs of the civil society as much as those of the MoH. Our findings suggested that some civil society actors felt that the DHIS2, the national RHIS does not always enable an appropriate representation of their organizations’ contribution to the health agenda. Furthermore, though many civil society respondents from our study characterized the mechanisms for data exchange between their organizations and the public sector as largely positive, there was a general sentiment that there are opportunities for improvement in terms of data access and sharing, particularly with regards to the DHIS2 platform. We recommend that the MoH, through the DSISS, explore mechanisms of accommodating the many inevitable routine data requests from the civil society with the understanding that making data accessible to a broad audience via a centralized system can be challenging. In its advocacy role, the civil society is an essential partner in the fight against HIV/AIDS, TB and malaria to ensure transparent and equitable service delivery [60, 61]. Ensuring that these actors have a voice in the decision-making related to country data needs is of primordial importance.

Building RHIS data systems capable of ensuring the continuity of clinical care and program responses will require improved coordination for the tracking of patients within and across programs. Study participants broadly perceived the double counting of patients who access HIV care services from different partners or facilities as a serious data quality problem that affects the credibility of RHIS data. To address this issue, it was clear that the development of unique personal identifiers enabling patient tracking and linkage across multiple health services should be a priority. Indeed, some have proposed that in order to develop data systems that can efficiently support the HIV response in the complex context of declining resources and multiple actors, it will be important to develop unique personal identifiers [62, 63]. The importance of investing in the adoption of unique patient identifiers is further reinforced when one considers the increasing integration of HIV health service delivery into other healthcare services such as TB and maternal and child health [64]. Nonetheless, as others have cautioned, with the development of unique identifiers, it is imperative to involve the civil society in the process to address concerns regarding the potential for confidentiality breaches and human rights abuses [62].

#### Build capacity and promote a culture of data quality assessment and information use

While some determinants of data use and data quality such as RHIS system design and some infrastructural factors may improve over time following technological and economic developments, overcoming many of the behavioral and organizational barriers we identified will require deliberate effort of building an information use culture. Building an information use culture where data is valued at all levels of the health system, from collection to use, is central to ensuring that RHIS processes are efficient and sustainable [31, 56]. Building such a culture over the long-term at a national level implies behavior change efforts at massive scale, a process that would undoubtedly take time. Yet, this is a necessary process.

Firstly, it will be important to encourage responsibility for data quality and data use at the local level. Engaging frontline data staff in facilities and communities to promote effective record keeping is certainly key. However, respondents in our study seemed to think that engaging district-level actors would be even more important. In a decentralized health system, the district authority occupies a uniquely strategic position in relation to program management and to monitoring and evaluation of integrated health services [65, 66]. In the Senegalese context, the CDMOs exercise significant authority over the health posts and health huts within their respective district. We therefore recommend bottom-up approaches that emphasize the role of the health district and the CDMO to create an information use culture. If the value of using quality data for local-decision making is impressed upon the CDMO, it is presumable that attitudinal changes and values would be transferred onto the CDMO’s subordinates because of the authority structure.

In many countries, though districts are typically mandated to analyze and act on the local data, literature suggests that many are rarely equipped to do so or simply lack the will [67]. Similarly, some study participants suggested that districts often failed to achieve optimal use of local RHIS data for local decision-making and often came short of their duty to conduct mandated quarterly DQAs. The MoH and its partners should seek to implement mechanisms for the institutionalization of district-level DQAs and data review meetings such that districts are incentivized to do this activity without central-level supervision. Here we define institutionalization as systems of norms (or behaviors) with strong but variable mechanisms of support and enforcement [68, 69]. Indeed, it has been previously argued that the institutionalization of data quality assessment may be the best preventive mechanism of ensuring good quality RHIS data [56].

For this to happen, the MoH will need to mobilize adequate and appropriate technical and financial resources. A crucial challenge will be balancing the level of attention and resources allocated to strengthening RHIS activities and systems against those allocated to other data types. Creating a sustainable information use culture means that the totality of information is appropriately valued. The MoH and its partners will therefore have to explore means of not only supporting RHIS activities, but also ensuring the sustainable conduct of existing research and survey activities (DHS) as well as investing in other new data generation activities such as the TB prevalence and risk mapping surveys.

### Strengths and limitations

This study is subject to several limitations that must be acknowledged. The findings presented in this study are based on opinions and experiences of key informants involved in central-level decision-making processes, rather than on empirical data from the different tiers of the Senegalese health system. Our study did not have the resources to include the perspectives of key informants from lower levels of the health system. Based on the consensus among respondents, and on evidence from literature [65] that data use issues are more pronounced at lower levels of the health system compared to central levels, future studies should focus on further clarifying data use determinants at that level. Importantly, the views of the key informants do not represent those of all experts in Senegal. Nonetheless, we note that the high levels of agreement among the different study participants on key issues may suggest reliability of the findings.

A strength of our study is our maximum variation sampling strategy that enabled us to consider a wide range of perspectives. Given the complexity of the programmatic landscape in Senegal, we found this sampling strategy to be most appropriate to both capture the different stakeholder experiences and have a more complete understanding of the context. The inclusion of stakeholders involved in HIV, TB and malaria control ensured that we were able to obtain the perspectives of a significant portion of the public health community. Nonetheless, we acknowledge that the participation of other programs such as the immunization and reproductive health programs would have further strengthened the research findings given their programmatic importance and the level of attention they receive in RHIS implementation. To have a more complete understanding of RHIS data use in Senegal, future studies should incorporate RHIS data from these programs in their analyses.

Another strength of the study was that participants were provided with a summary of the interview findings to ensure the credibility of the findings.

We note that some of the issues discussed by this research may be time-restricted and Senegal-specific thus limiting the transferability of research findings to other contexts. In the current period, for instance, health data retention during strikes seems to play an important role in determining data quality and data use in Senegal. This factor may not be relevant in other settings so our findings may not be generalizable to other contexts without further investigation.

## Conclusion

Our study adds new evidence confirming that the use of routine data for strategic decision-making processes is ultimately linked to the availability and quality of the data. When fully leveraged, RHIS may provide timely and granular data capable of driving programmatic action at wide geographic scales. However, achieving the full potential of RHIS in Senegal will require addressing complex challenges particularly at lower levels of the health system. In the short term, it will be important to minimize data retention strikes and maintain uninterrupted surveillance activities by addressing the different structural challenges faced by healthcare staff working at the operational levels of the health system. Coordinated action at multiple levels of the health system will be required to institutionalize district-level data quality reviews while strengthening feedback and supervision mechanisms. Further coordination efforts will be required to harmonize data collection tools particularly at the community-level and simplify data collection processes. It is likely more effective to pursue the collection of fewer, more meaningful and complementary data than to invest resources in collecting an abundant array of data that will not be used or perceived as useful. On a more long-term scale, we urge the strengthening of inclusive partnerships that emphasize the role of data producers from the private sector and the civil society. This increased participation will not only contribute to increased data completeness and representativeness, but presumably also a sense of shared responsibility to improve data quality and data use. Ultimately, this will be important in promoting accountability and the building of a robust data use culture. Given the complex programmatic context within which routine data are produced and used in Senegal, the challenge for the Ministry of Health will be figuring out how to best shape the environment within which the multitude of stakeholders operates.

## Data Availability

The datasets generated and/or analyzed in the current study are not publicly available due to privacy concerns but are available from the corresponding author upon reasonable request.

## Abbreviations

CDMO: Chief District Medical Officer
CHW: Community Health Worker
CNERS: Senegal National Ethical Committee for Health Research
CSO: Civil Society Organization
DHIS2: District Health Information System Version 2
DHS: Demographic and Health Survey
DM: Decision-maker
DSISS: Division of Social and Health Information Systems
JHU: Johns Hopkins University
MOH: Ministry of Health
MP: Mid-level personnel
NGO: Non-Governmental Organization
NMCP: National Malaria Control Program
RDT: Rapid diagnostic test
RHIS: Routine Health Information system
SPA: Service Provision Assessment survey
UCAD: Université Cheikh Anta Diop de Dakar
WHO: World Health Organization
